# Tideglusib Rescues Neurite Pathology of SPG11 iPSC Derived Cortical Neurons

**DOI:** 10.3389/fnins.2018.00914

**Published:** 2018-12-06

**Authors:** Tatyana Pozner, Annika Schray, Martin Regensburger, Dieter Chichung Lie, Ursula Schlötzer-Schrehardt, Jürgen Winkler, Soeren Turan, Beate Winner

**Affiliations:** ^1^Department of Stem Cell Biology, Friedrich–Alexander University Erlangen–Nürnberg, Erlangen, Germany; ^2^Department of Neurology, Friedrich–Alexander University Erlangen–Nürnberg, Erlangen, Germany; ^3^Department of Molecular Neurology, Friedrich–Alexander University Erlangen–Nürnberg, Erlangen, Germany; ^4^Institute of Biochemistry, Friedrich–Alexander University Erlangen–Nürnberg, Erlangen, Germany; ^5^Department of Ophthalmology, Friedrich–Alexander University Erlangen–Nürnberg, Erlangen, Germany; ^6^Center of Rare Diseases Erlangen, Friedrich–Alexander University Erlangen–Nürnberg, Erlangen, Germany

**Keywords:** induced pluripotent stem cell, neuronal culture, SPG11, tideglusib, GSK3β inhibitor, hereditary spastic paraplegia, CRISPR knock-out

## Abstract

Mutations in SPG11 cause a complicated autosomal recessive form of hereditary spastic paraplegia (HSP). Mechanistically, there are indications for the dysregulation of the GSK3β/βCat signaling pathway in SPG11. In this study, we tested the therapeutic potential of the GSK3β inhibitor, tideglusib, to rescue neurodegeneration associated characteristics in an induced pluripotent stem cells (iPSCs) derived neuronal model from SPG11 patients and matched healthy controls as well as a CRISPR-Cas9 mediated SPG11 knock-out line and respective control. SPG11-iPSC derived cortical neurons, as well as the genome edited neurons exhibited shorter and less complex neurites than controls. Administration of tideglusib to these lines led to the rescue of neuritic impairments. Moreover, the treatment restored increased cell death and ameliorated the membranous inclusions in iPSC derived SPG11 neurons. Our results provide a first evidence for the rescue of neurite pathology in SPG11-HSP by tideglusib. The current lack of disease-modifying treatments for SPG11 and related types of complicated HSP renders tideglusib a candidate compound for future clinical application.

## Introduction

Hereditary spastic paraplegias (HSPs) are a heterogeneous group of motor neuron disorders. Clinically, HSP manifests with progressive lower limb spasticity and weakness due to axonopathy of corticospinal motor neurons and ascending dorsal columns. Up to date, more than 70 distinct genetic loci (Spastic Paraplegia Gene SPG1-SPG79) and mutations in more than 50 genes have been identified in HSP patients (Lo Giudice et al., 2014; Novarino et al., 2014). HSPs can be inherited in an autosomal dominant, autosomal recessive (AR), and, rarely, in an X-linked manner. Mutations in SPG11 are the most common genetic cause of AR complicated HSP. Apart from spastic paraparesis, SPG11 patients present with additional phenotypes that in the majority of cases include cognitive impairment, thin corpus callosum (TCC), neuropathy, and amyotrophy (Lo Giudice et al., 2014). Interestingly, mutations in *SPG11* encoding spatacsin, were also found in other motor neuron diseases such as AR juvenile-onset amyotrophic lateral sclerosis (ALS5) and AR Charcot-Marie-Tooth disease (Montecchiani et al., 2016; Orlacchio et al., 2016). This indicates an important function of *SPG11* in a variety of neuronal subtypes and shows that spatacsin causes multi system neurodegeneration.

Until now, more than 100 mutations in the 40-exon long SPG11 have been described. The majority of mutations result in premature stop codons, causing nonsense mediated RNA decay and/or truncated proteins. This implies a loss of function mechanism (Stevanin et al., 2013). SPG11 encodes spatacsin (a 2443 aa protein; ∼280 kDa), a potential transmembrane protein (Paisan-Ruiz et al., 2008) that has been implicated in axonal maintenance (Pérez-Brangulí et al., 2014). A number of studies have shown that the protein is linked to the autophagic-lysosomal machinery (Chang et al., 2014; Renvoise et al., 2014; Varga et al., 2015; Branchu et al., 2017). In addition, siRNA mediated knock-down of SPG11, led to reduced neurite complexity of mouse dissociated cortical neurons (Pérez-Brangulí et al., 2014). Moreover, membrane-bound structures were observed within the processes of SPG11 patient, induced pluripotent stem cells (iPSC) derived cortical neurons (Pérez-Brangulí et al., 2014). This observation has been related to alterations in the transport of organelles, specifically anterograde transport and a lack of synaptic vesicle movement in SPG11 neurons (Pérez-Brangulí et al., 2014). Additionally iPSC-derived neural progenitor cells (NPCs) described a prominent neurodevelopmental defect. Reduced NPC proliferation was mediated by increased GSK3β activity followed by impairment of β-catenin signaling pathway (Mishra et al., 2016). Interestingly, administration of the specific GSK3β inhibitor tideglusib rescued the observed NPC proliferation defect (Mishra et al., 2016).

In the present study, we asked, whether tideglusib might not only improve proliferation, but might also have a positive effect on neurite pathology. We evaluated the effect of tideglusib on SPG11 patients’ neurons. After confirming the presence of neurite pathology in iPSC derived cortical neurons from SPG11 patients and genome edited lines, we here established a treatment regimen for differentiated cortical neurons. By analyzing neuronal morphology and survival, we demonstrate that tideglusib is capable to reduce these neuronal impairments.

## Materials and Methods

### Patients

The patients included in this study (*n* = 3; Table 1) were female Caucasians with genetically confirmed compound heterozygous mutations in SPG11 (Hehr et al., 2007; Bauer et al., 2009; Pérez-Brangulí et al., 2014). SPG11-1 and SPG11-2 are sisters with a heterozygous nonsense mutation at c.3036C>A/p.Tyr1012X in exon 16 and a c.5798delC/p.Ala1933ValfsX18 mutation in exon 30. SPG11-3 has a heterozygous nonsense mutation at c.267G>A/p.Trp89X in exon 2 and a splice site mutation 1457-2A>G in intron 6. All patients were severely affected, also indicated by high scores on the Spastic Paraplegia Rate Scale (33–39 out of max. of 52; Schüle et al., 2006). All patients have a TCC accompanied by cognitive impairment, white matter lesions, cortical atrophy, as well as muscle wasting and motor-sensory neuropathy (Hehr et al., 2007). The controls (CTRL1; CTRL2) were age matched healthy Caucasian females with no previous history of movement or neurological disorders.

**Table 1:** Clinical characterization of SPG11 patients and controls.

	SPG11-1	SPG11-2	SPG11-3	CTRL-1	CTRL-2
SPG11 mutations	Exon 16: c.3036C>A Exon 30: c.5798delC	Exon 16: c.3036C>A Exon 30: c.5798delC	Exon 2: c.267G>A Intron 6: c.1457-2A > G	-	-
Sex	Female	Female	Female	Female	Female
Age at onset/age at examination (years)	24/46	20/40	31/50	–/45	–/28
SPRS (0–52)	44	37	36	0	0
Cognitive impairment	+	+	+	-	-
Wheelchair dependency	+	+	+	-	-
MRI abnormalities	Cortical atrophy, WML, TCC	Cortical atrophy, WML, TCC	Cortical atrophy, WML, TCC	-	-
iPSC clones (*n* = 2 per line)	SPG11-1a, SPG11-1b	SPG11-2a, SPG11-2b	SPG11-3a, SPG11-3b	CTRL-1a, CTRL-1b	CTRL-2a, CTRL-2b

*Patients, SPG11-1, SPG11-2, SPG11-3; controls, CTR-1, CTRl-2; SPRS, spastic paraplegia rating scale; TCC, thin corpus callosum; WML, white matter lesion; iPSC, induced pluripotent stem cells*.

### iPSC Derivation

Fibroblasts from SPG11 patients and controls were reprogrammed with retroviral transduction using the four Yamanaka factors (Klf4, c-Myc, Oct4, and Sox2) as previously described (Pérez-Brangulí et al., 2014). The Institutional Review Board approval (Nr. 4120: Generierung von humanen neuronalen Modellen bei neurodegenerativen Erkrankungen) and informed and written consent forms are on file at the Movement Disorder Clinic of the Department of Molecular Neurology, Universitätsklinikum Erlangen (Erlangen, Germany). All iPSC lines were screened for pluripotency and had a stable karyotype using the G-banding chromosomal analysis (data not shown). The SPG11 mutations were confirmed in the patient-derived lines. Two iPSC lines were used for each patient and control.

### Targeted SPG11 Knock-Out With the CRISPR/Cas9 System

To generate the SPG11 knock-out line (cSPG11) we targeted exon 1 of SPG11 (Figure 4A). The single gRNAs (sgRNA) (Sigma-Aldrich) were chosen with the CRISPOR web-tool^1^ and cloned into the pX330 plasmid expressing SpCas9 (Addgene plasmid #42230) and the sgRNA according to the guidelines of the Zhang lab^2^. The cutting efficiency of the sgRNAs was evaluated in transfected 293T cells, by T7 endonuclease-mediated detection of insertions/deletions (indels) as previously described (Turan et al., 2016). The highly efficient sgRNA with an off-target score of 92 (sequence at Supplementary Figure S3) was chosen for nucleofection of a human embryonic stem cell line (hESC; HUES6). All the experiments with HUES6 were conducted according to the German Stem Cell Act (RKI, 63. Genehmigung to BW). Forty-eight hours after nucleofection, cells were single cell sorted by flow cytometry, expanded and validated for homozygous knock-out by T7-endonuclease assay (Supplementary Figure S2A), sequencing (LGC genomics) and TIDE genotyping analysis^3^. The positive clones and isogenic control (clone that underwent the genome editing process but had no mutations) were evaluated for exonic off-target mutagenesis by PCR-mediated sequencing of potential off-target sites of 5 candidate genes (selected with CRISPOR) (Supplementary Figure S3A). The genome edited lines were cultured on Matrigel (BD Biosciences) in mTeSR1 medium (Stem Cell Technologies). Detailed experimental scheme appears in Figure 4B.

### Neuronal Differentiation and Tideglusib Treatment

**FIGURE 1:**
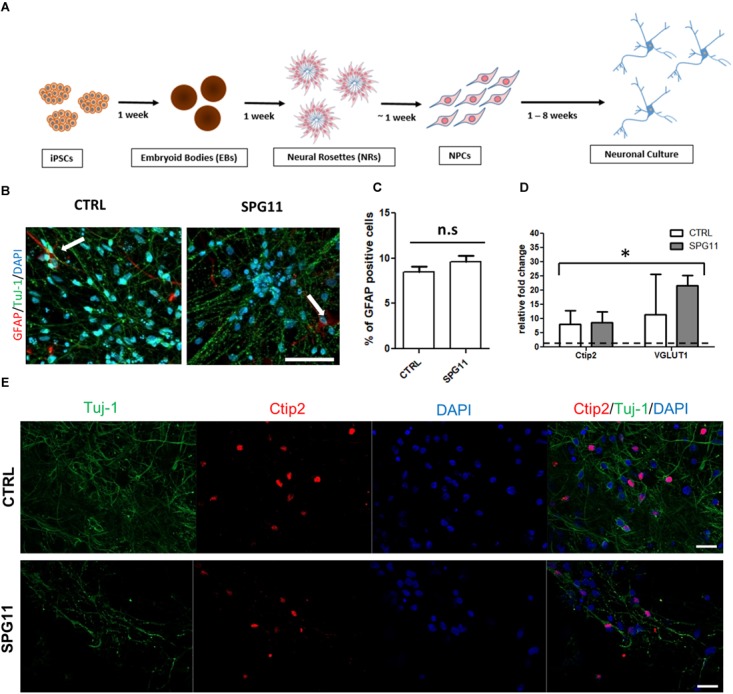
Neuronal differentiation and characterization **(A)** Schematic representation of the differentiation of iPSCs into neuronal cultures. **(B)** Representative images of CTRL (left; CTRL-1a) and SPG11 (right; SPG11-1b) 4 weeks differentiated neurons stained for GFAP and Tuj-1. **(C)** Quantification of percentage of GFAP^+^/DAPI^+^ cells in CTRLs and SPG11. **(D)** qPCR data of 4 weeks differentiated neurons. RNA from four different iPSCs clones was used as negative control and it is indicated as a dashed line at onefold change. **(E)** Representative images Ctip2 (red) and Tuj-1 (green) of CTRL and SPG11. Quantifications represent pooled data of CTRLs (CTRL-1, CTRL-2) and SPG11 (SPG11-1, SPG11-2, SPG11-3) neurons. iPSCs, induced pluripotent stem cells; NPCs, neural progenitor cells. Scale bar = 50 μm in **(B)** and 20 μm in **(E)**. ^∗^*P* < 0.05.

The generation of NPCs and subsequent differentiation from pluripotent stem cells into neuronal cells was conducted as described previously (Havlicek et al., 2014; Figure 1A). Briefly, pluripotent stem cells were cultured with mTeSR1 medium (Stemcell Technologies) and passaged using Gentle Cell Dissociation Reagent (Stemcell Technologies). For the generation of free floating embryoid bodies (EBs), they were incubated for 45 min at 37°C with Collagenase IV and transferred to ultra-low attachment plates (Corning) for 1 week. Afterwards, the EBs were transferred to polyornithine-laminin coated plates, followed by manual collection of neural rosettes. Rosettes were dissociated using TrypLE^TM^ Express and transferred to proliferation media containing FGF_2_. For the induction of neuronal differentiation, the cells were cultured in N2/B27 media supplemented with the neurotrophins BDNF and GDNF, cAMP, and ascorbic acid. Tideglusib (Selleckchem; diluted in DMSO) was administered at the concentration of 1 μM to 1 week differentiated neurons and was applied twice a week, during media changes, for a period of 3 weeks (for details see paradigm Supplementary Figure S1).

### Immunofluorescence and Image Analysis

For stainings, 100,000 NPCs were plated on 24-well plates with polyornithine-laminin coated glass cover slips and treated according to the paradigm in Figure 1A. To quantify neuronal cell death, PFA-fixed cells were pretreated with acetic acid and ethanol at a 1:2 ratio at -20°C for 5 min. Afterward, the cells were stained with anti βIII-tubulin (Tuj-1; 1:500; BioLegend) and anti-cleaved-caspase3 (cCasp3; 1:500; Cell Signaling Technology) antibodies and mounted on microscope slides. Triplicate coverslips were used for each cell line and three random images per coverslip with comparable cell densities (40×) were acquired using Observer.Z1 fluorescence microscope (Zeiss). Representative images were obtained with LSM-780 confocal microscope setups (Carl Zeiss). For additional stainings, anti-glial fibrillary acidic protein (1:500; DAKO), and anti-Ctip2 (1:300; Abcam) antibodies were used. The quantifications were conducted with the cell counter plugin of Fiji software (Schindelin et al., 2012).

### Gene Expression Analysis

The RNA was extracted using RNeasy kit (Qiagen) according to the manufacturer’s instructions. A total of 500 ng RNA was reverse-transcribed into cDNA in 20 μl reaction solution by QuantiTect Reverse Transcription Kit (Qiagen). Subsequently, 1 μl of cDNA was used for real-time polymerase chain reaction (qPCR). The qPCR program definitions were as follows: 95°C for 10 min, followed by 40 cycles at 95°C for 15 s and 60°C for 1 min and 1 cycle at 95°C for 15 s, 60°C for 30 s and 95°C for 15 s. The primers are listed in Supplementary Table S1.

### Neurite Length and Complexity Analysis

Neurons derived from pluripotent stem cells were cultured in 24-well plates, on top of polyornithine-laminin coated glass coverslips. In order to allow visualization of single neurons, on day 26 the cells were transfected with pEF1-dTomato using Lipofectamine, 2000 (Invitrogen, reagent:DNA ratio 2:1). The cells were fixed 48 h after transfection with 4% paraformaldehyde (PFA) in phosphate-buffered saline (PBS). The cells were stained for neuronal marker expression using the anti-βIII-tubulin antibody on coverslips (Tuj-1; 1:500; BioLegend), and the coverslips were mounted on slides. Images were captured with a 10× objective lens using the TILE function of the Zen Pro Software (Zeiss) on the Observer.Z1 fluorescence microscope (Zeiss). Semi-automated tracing of individual transfected βIII-tubulin positive cells was performed using the NeuronJ plugin of Fiji (Schindelin et al., 2012). A minimum of 36 cells per patient/control and 18 cells for genome edited lines were analyzed for the number of neurites and total neurite length. Furthermore, semi-automated Sholl analysis was carried out at predefined 50 μm intervals from the soma using the Fiji software.

### Electron Microscopy

Transmission electron microscopy was performed as described previously (Schlotzer-Schrehardt et al., 2012; Havlicek et al., 2014). Briefly, the neurons were fixed in 2.5% glutaraldehyde in 0.1 M phosphate buffer. Subsequently, cells were post-fixed in 2% buffered osmium tetroxide and dehydrated in graded alcohol concentrations. Afterward, the cells were embedded in epoxy resin. Horizontal sections were stained with uranyl acetate and lead citrate. Finally, the sections were examined using a transmission electron microscope (LEO 906E; Carl Zeiss Microscopy).

### Statistical Analysis

All statistical analyses were performed using IBM SPSS statistics software (version 23). The Student two tailed *t*-test for unpaired variables was applied when comparing the means between two groups. For comparison of more than three groups, one-way ANOVA followed by Bonferroni *post hoc* test was applied. *P*-values ≤ 0.05 were considered statistically significant. Unless indicated otherwise, all data are shown as mean ± SEM.

## Results

### Characterization of iPSC Derived Neurons

The neuronal differentiation (described in Figure 1A) yields in cortical neurons expressing a majority of Tuj-1 positive cells with astrocytes comprising less than 10% of the cellular population (Figures 1B,C). There was no significant difference in the amount of astrocytes between SPG11 lines and controls (Figure 1C).

Immunofluorescence stainings as well as qPCR analysis confirm the neuronal cortical identity of the iPSC derived cells (Figures 1D,E). Thus, both controls and SPG11 cell lines exhibited a significantly higher transcript level of the deep layer marker Ctip2 and the vesicular glutamate transporter 1 (VGLUT1) compared to iPSC lines (Figure 1D). The gene expression levels did not differ between patients and controls (Figure 1D).

### Rescue of SPG11 Neurite Outgrowth Abnormalities by Tideglusib Treatment

**FIGURE 2:**
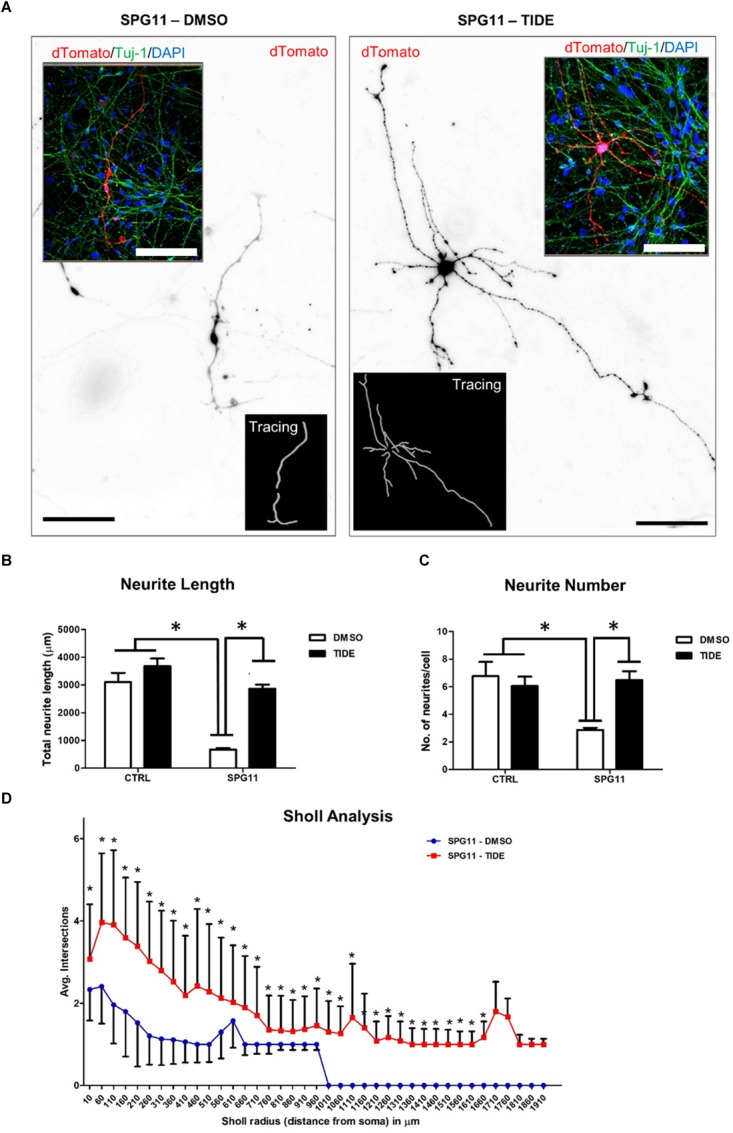
Rescue of neurite outgrowth abnormalities of SPG11 patients by tideglusib treatment. **(A)** Representative image of non-treated (left) and tideglusib treated (right) SPG11 neurons transfected with pEF1-dTomato. Neurite tracings are shown in the inserts. **(B)** The total neuritic length is significantly decreased in SPG11 neurons and it is recovered following tideglusib treatment. **(C)** The average number of SPG11 neurites is significantly decreased and it is recovered following tideglusib treatment. **(D)** Sholl analysis confirms a significant increase in neurite number following tideglusib treatment. Quantifications in C, D represent pooled data of CTRLs (CTRL-1, CTRL-2) and SPG11 (SPG11-1, SPG11-2, SPG11-3) iPSC-derived-neurons. Scale bar = 100 μm. Data shown as means ± SEM. ^∗^*P* ≤ 0.05.

We first evaluated the neurite morphology of SPG11 iPSC derived neuronal cultures and measured neuritic length and the number of neurites per cell, indicative of neuritic complexity (Figure 2A). Compared to CTRLs, neurite length was significantly decreased by 78% in SPG11 (669.44 ± 50.34 μm in SPG11, compared to 3103.48 ± 328.04 μm in CTRL; *P* ≤ 0.05; Figure 2B). Moreover, neurite number was significantly reduced (2.87 ± 0.16 in SPG11, compared to 6.76 ± 1.0 in CTRL; *P* ≤ 0.05; Figure 2C). These results are in agreement with the previously described neuritic impairment in SPG11 iPSC derived neuronal cultures (Pérez-Brangulí et al., 2014). We next tested the effect of the chronic administration of 1 μM tideglusib. The rationale for this dose was obtained from previous experiments, which revealed severe toxicity at a higher dose (5 μM; data not shown). We applied the compound twice a week for a period of 3 weeks until neurons reach maturity (see paradigm Supplementary Figure S1). Interestingly, chronic administration of tideglusib was able to significantly rescue neurite length (2856.86 ± 151.65 μm in SPG11 treated with tideglusib, compared to 3103.48 ± 328.04 μm in CTRL; *P* ≤ 0.05; Figure 2B). Moreover, neurite number was restored to a level which was virtually comparable to CTRL (6.48 ± 0.64 in SPG11 treated with tideglusib, compared to 6.76 ± 1.04 in CTRL; *P* ≤ 0.05; Figure 2C).

In order to further elaborate the effect of the chronic administration of tideglusib on neurite complexity, Sholl analysis was performed. The number of intersections of neurites at various radial distances from the cell soma was measured and revealed a significant increase in neuritic arborization of SPG11 patient derived neurons upon tideglusib treatment (Figure 2D). This effect was significant for all distances’ measures (distances from soma from 10 μm to 1660 μm, *P* ≤ 0.05).

### Rescue of Neurodegeneration Associated Characteristics in SPG11 iPSC-Derived Neurons by Tideglusib Treatment

**FIGURE 3:**
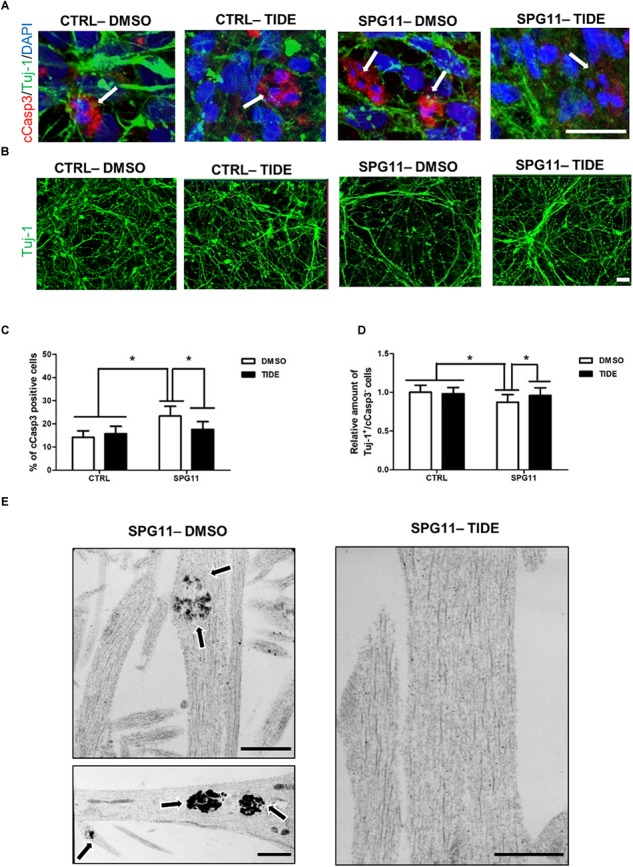
Neurodegeneration associated impairments in SPG11 neurons are rescued by tideglusib treatment. **(A)** Merged confocal images of SPG11 and CTRL neurons generated from iPSCs and co-immunostained for βIII-tubulin (Tuj-1) and cleaved-caspase3 (cCasp3). Scale bar = 20 μm. **(B)** Representative images of TUJ-1 stained SPG11 and control neurons. **(C)** Elevated cCasp3 levels in SPG11 neurons is rescued by tideglusib treatment. **(D)** Reduced level of Tuj-1 is rescued by tideglusib treatment. **(E)** Ultrastructural analysis of the neurites of non-treated (left) and tideglusib treated (right) SPG11 4-week-differentiated-neurons reveals that the membranous inclusion bodies (indicated by arrows) in SPG11 neurons are reduced in number and size following the treatment. Quantifications in **(C,D)** represent pooled data of CTRLs (CTRL-1, CTRL-2) and SPG11 (SPG11-1, SPG11-2, SPG11-3) iPSC-derived-neurons. Scale bar = 20 μm in **(A,B)** and 1 μM in **(E)**. ^∗^*P* < 0.05.

We next asked, whether treatment with tideglusib also has an impact on neural cell death. We quantified the number of cCasp3/Tuj1 double positive cells within the different groups (Figure 3A). A significantly higher amount of neuronal cell death was present in non-treated SPG11 neurons (23.34% ± 04.22% cCas3^+^ cells in SPG11, compared to 14.17% ± 2.80% cCas3^+^ cells in CTRL; *P* ≤ 0.05; Figure 3C). In addition, the relative survival rate was lower in SPG11 neurons compared to control (0.87 ± 0.09 Tuj-1^+^/ cCas3^-^ cells in SPG11 treated with tideglusib, compared to 1.0 ± 0.09 Tuj-1^+^/cCas3^-^ cells in CTRL; *P* ≤ 0.05; Figures 3B,C). The treatment decreased the number of cCas3^+^ cells to CTRL levels (15.68 ± 2.26% cCas3^+^ cells in SPG11 treated with tideglusib, compared to 14.17 ± 2.80% cCas3^+^ cells in CTRL; *P* ≤ 0.05; Figures 3A,C). Similarly, the compound was also able to increase the survival rate of SPG11 neurons (0.96 ± 0.09 Tuj-1^+^/cCas3^-^ cells in SPG11 treated with tideglusib, compared to 1.0 ± 0.09 Tuj-1^+^/cCas3- cells in CTRL; *P* ≤ 0.05; Figures 3B,C) to a level which did not significantly differ from CTRL (*P* ≥ 0.05 according to Bonferroni test; Figure 3D). We next sought to analyze the impact of tideglusib on neurite ultrastructure.

The ultrastructural analysis of SPG11 iPSC derived 4-weeks differentiated neurons revealed that the neurites accumulate membrane-bound inclusion bodies measuring 0.3–1.5 μm in diameter with electron-dense contents. Interestingly, tideglusib treatment reverted this phenotype and led to an elimination of these abnormal structures (Figure 3E).

### CRISPR/Cas9 Mediated Generation and Characterization of SPG11 Knock-Out Line

**FIGURE 4:**
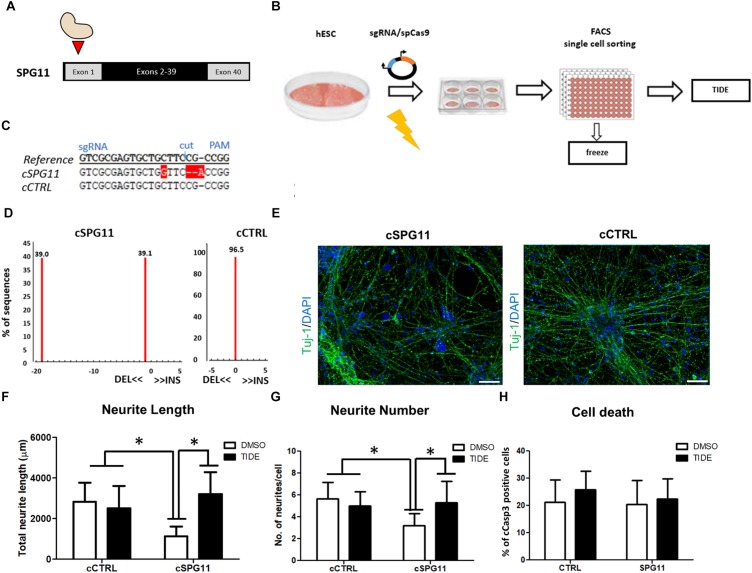
CRISPR/Cas9 genome edited hESC-derived-neurons recapitulate the neuritic phenotypes of patient iPSC-derived-neurons. **(A)** Strategy for SPG11 knock-out by targeting exon 1 of *SPG11*. **(B)** Experimental scheme for CRISPR/Cas9 mediated genome editing of hESCs. hESCs were nucleofected with 5 μg of pX330 plasmid expressing SpCas9 and the sgRNA. After 48-h, the cells were single cell-sorted into 96-well plate. Subsequently, emerging colonies were split in 1:2 ratio. Half of the split was used for genomic DNA generation. The rest was transferred into a new 96-well plate for cryopreservation in –80°C. For identification of positive clones, PCR products were sequenced, further expanded and genotyped by Tracking Indels by Decomposition (TIDE; https://tide.nki.nl/) web-tool. **(C)** Sequence comparison between reference non-nucleofected HUES6 cell line, a positive, genome edited clone (cSPG11) and a control that underwent the genome editing process, but had no mutations (cCTRL). **(D)** TIDE analysis of cSPG11 and cCTRL. **(E)** Representative images of the genome edited lines stained for Tuj-1 (green). **(F)** The total neuritic length is significantly decreased in cSPG11 neurons and it is recovered following tideglusib treatment. **(G)** The average number of SPG11 neurites is significantly decreased and is recovered following tideglusib treatment. **(H)** Quantification of cCasp3^+^ cells reveals no significant difference between cSPG11 and cCTRL. Data shown as means ± SD. Scale bar = 50 μm. ^∗^*P* < 0.05. FACS = Fluorescence-activated cell sorting.

Sequencing analysis of HUES6 line transfected with a plasmid containing Cas9 and gRNA targeting exon 1 of SPG11 revealed one clone with out-of-frame mutation (cSPG11, Figures 4C,D). According to TIDE analysis, the clone was positive for out-of-frame mutation in both alleles (Figures 4C,D). An additional clone, that underwent the genome editing process, but had no mutations (cCTRL; Figure 4D), was chosen as isogenic control. Both cSPG11 and cCTRL were negative for off-target activity (Supplementary Figures S3A,B). The reduction of spatacsin in cSPG11 confirms the efficiency of the genome editing strategy (Supplementary Figure S2B). The residual amount of the protein can be attributed to the lack of specific and reliable antibodies for spatacsin detection. Genome edited pluripotent stem cells were differentiated according to the paradigm in Figure 1A and were positive for Tuj1 (Figure 4E) with astrocytes levels that, similarly to iPSC-derived neuronal cultures, were lower than 10% and did not differ between cSPG11 and cCTRL (Supplementary Figure S2C).

#### Rescue of Neurite Impairments in the Genome Edited Line

Interestingly, the SPG11 genome edited line (cSPG11) recapitulates the observations from the patients’ iPSC derived neurons (Figures 4F,G). Thus, the neurites of cSPG11 hES derived neuronal cultures are significantly shorter (1115.99 ± 490.04 μm in cSPG11, compared to 2816.25 ± 948.44 μm in cCTRL; *P* ≤ 0.05; Figure 4F) and fewer (3.22 ± 1.09 in cSPG11, compared to 5.66 ± 1.50 in cCTRL; *P* ≤ 0.05; Figure 4G) compared to isogenic control (cCTRL). Similarly to the rescue of the patients’ lines, tideglusib administration rescued the neurite length (3211.18 ± 1080.52 μm in cSPG11 treated with tideglusib, compared to 2816.25 ± 948.44 μm in cCTRL; *P* ≤ 0.05; Figure 4F) and complexity (5.33 ± 1.93 in cSPG11 treated with tideglusib, compared to 5.66 ± 1.50 in cCTRL; *P* ≤ 0.05; Figure 4G).

There was no evident change in the levels of cell death between the genome edited control (cCTRL) and SPG11 knock-out (cSPG11) lines (Figure 4H).

## Discussion

Our findings indicate a beneficial effect of tideglusib on both cellular morphology and survival of SPG11-iPSCs-derived neurons. This is the first indication of an effective therapeutic approach applied to human SPG11 differentiated neurons.

First, our data confirm the findings from a previous study (Pérez-Brangulí et al., 2014) that SPG11 neurons present with impaired neuritic length and decreased number of neurites, indicative of reduced neuronal complexity. Our study was able to rescue these impairments by administration of the irreversible GSK3 inhibitor, tideglusib, to neuronal cells. In addition, we were able to recapitulate virtually similar neuritic impairments, followed by tideglusib rescue in CRISPR/Cas9 mediated SPG11 knock-out line. Previous findings revealed that SPG11 NPCs exhibit dysregulation of GSK3β/βCat signaling pathway (Mishra et al., 2016). The data of our current study indicate that this pathway has a prominent role not only during neural development, but also in mature neurons. We are able to show, that in SPG11 tideglusib has a significant role in rescuing the neuritic morphology.

Increased activity of GSK3β has been implicated in neurodegeneration, particularly in Alzheimer’s Disease (AD). Animal models overexpressing GSK3β exhibit one of the main AD hallmarks – increased tau phosphorylation (Brownlees et al., 1997; Lucas et al., 2001), whereas overexpression of GSK3β in the dentate gyrus leads to neurodegeneration of this area (de Barreda et al., 2010). Tau hyper-phosphorylation, elevated amyloid levels and activation of GSK3β are also present in AD-iPSC derived neurons (Ochalek et al., 2017). Conversely, inhibition of GSK3β leads to reduction of β-amyloid production (Rockenstein et al., 2007) and its toxicity (Koh et al., 2008), reduction of phosphorylated tau in cultured neurons (Zhang et al., 2011) and improves learning and memory in AD mouse models (Farr et al., 2016). Although the onset and patterns of cognitive symptoms and cortical atrophy present in SPG11 patients are markedly different from those observed in AD patients, our data suggest that GSK3β over-activation may be a common molecular denominator of both diseases.

We next found that tideglusib treatment was able to rescue the increased cell death of SPG11 neurons. The potential of tideglusib to diminish apoptosis in SPG11 was first revealed in a study where tideglusib administration to SPG11-NPCs improved their survival and proliferation (Mishra et al., 2016). Here, we extended the therapeutic value of tideglusib to neurons by showing for the first time, that differentiating neurons are also responsive to its effects. Our results are compatible with the data indicating the important role of GSK3β in the regulation of apoptosis (Jacobs et al., 2012). In addition, it has been shown that GSK3β inhibition leads to neuroprotective effects (Culbert et al., 2001).

Moreover, in rat cerebellar granule neurons cultures GSK3β inhibition lead to increase of neuronal survival (Song et al., 2010), whereas in a mouse model of CNS injury it promoted axonal regeneration (Song et al., 2010; Guo et al., 2016). Overall, these studies conclude that GSK3β inhibition has a beneficial effect on mature neurons which also provides an explanation to the observed rescue of SPG11 neurodegenerative phenotypes by tideglusib.

Interestingly, SPG11 knock-out line showed no difference in cell death, and the number of cCas3 positive cells was not changed following tideglusib treatment. This result may arise from the vast heterogeneity and lack of correlation between genotype and phenotype in *SPG11*. Thus, in addition to HSP type 11, mutations in *SPG11* can lead to autosomal recessive juvenile amyotrophic lateral sclerosis and Charcot-Marie-Tooth disease (Montecchiani et al., 2016). This observation indicates the importance of the use of patient specific lines for disorders with complex genotypes.

An additional important observation is that tideglusib treatment leads to reduction of membranous inclusion bodies in SPG11 neurites. The possibility to reduce this impairment emphasizes the potency of tideglusib as a putative therapeutic compound.

Overall, our results increase the clinical relevance of the compound tideglusib and its potential to serve as a therapeutic agent for SPG11. Furthermore, they contribute to the establishment of iPSCs as a potent tool for disease modeling and drug testing. The fact that tideglusib has been approved by FDA and has been tested in clinical trials of Alzheimer’s Disease, Autism Spectrum Disorder and Myotonic Dystrophy renders a potential availability also for SPG11 patients. Future studies that will utilize the advanced cerebral organoid models and delve into the mechanistic changes occurring after compound administration are essential for providing a robust basis for clinical implementation.

## Author Contributions

BW and JW participated in the conceptualization. ST conceived the methodology. DCL interpreted the findings and approved the final manuscript. TP, AS, and US-S performed the experiments and data analysis. BW, TP, and MR wrote and edited the manuscript and figures.

## Conflict of Interest Statement

The authors declare that the research was conducted in the absence of any commercial or financial relationships that could be construed as a potential conflict of interest.

## Footnotes

1 http://crispor.tefor.net/

2 https://www.addgene.org/crispr/zhang/

3 https://tide.nki.nl/
