# Supplementary material for: Tideglusib Rescues Neurite Pathology of SPG11 iPSC Derived Cortical Neurons
Source: Front Neurosci. 2018 Dec 6;12:914. doi: 10.3389/fnins.2018.00914 (PMC6291617; doi:10.3389/fnins.2018.00914)
Supplement: Supplementary file 1 [file Data_Sheet_1.docx]

Supplements for manuscript:

**Tideglusib rescues neurite pathology of SPG11 iPSC derived cortical neurons**

Authors: Tatyana Pozner^1*^, Annika Schray^1*^, Martin Regensburger^1, 2, 3^, D. Chichung Lie^4^, Ursula Schlötzer-Schrehardt^5^, Jürgen Winkler^3, 6^, Soeren Turan^1,4^, Beate Winner^1,6^


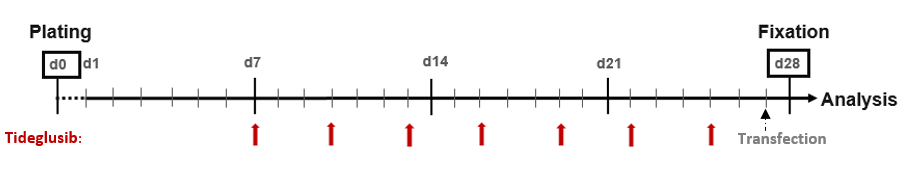


Fig. S1. **Tideglusib treatment paradigm.** On d0 NPCs were plated on polyornithine-laminin coated cover-slips in neuronal differentiation medium. Tideglusib administration was started on d7, and the compound was applied twice a week for a period of 3 weeks, during media changes. The cells were transfected with pEF1-dTomato (indicated by dashed arrow) 48h prior to fixation for further analysis. d - day.


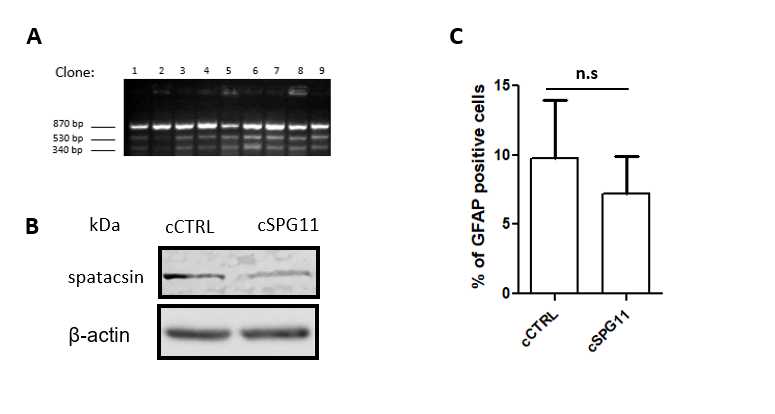


Fig. S2 Validation of the SPG11 knock out (cSPG11) and respective control (cCTRL) genome edited lines. (A) T7 endonuclease assay for validation of Cas9-mediated SPG11 knockout in clones that were isolated by single cell sorting. (B) Validation of spatacsin protein expression in the genome edited lines. (C) Quantification of percentage of GFAP positive cells in the genome edited lines. Data: mean ± SEM.


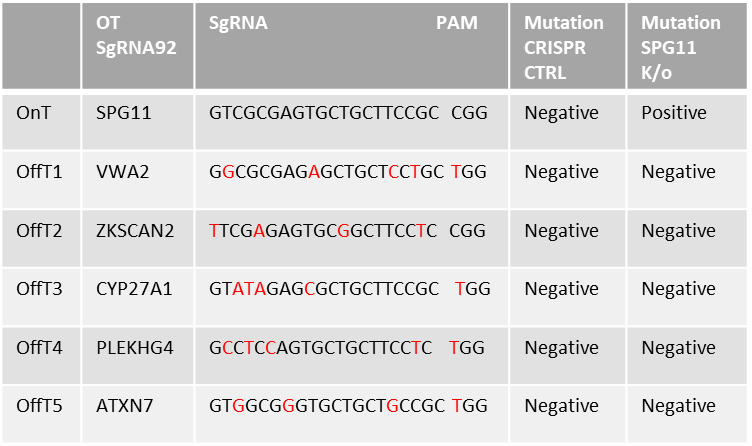


**A**


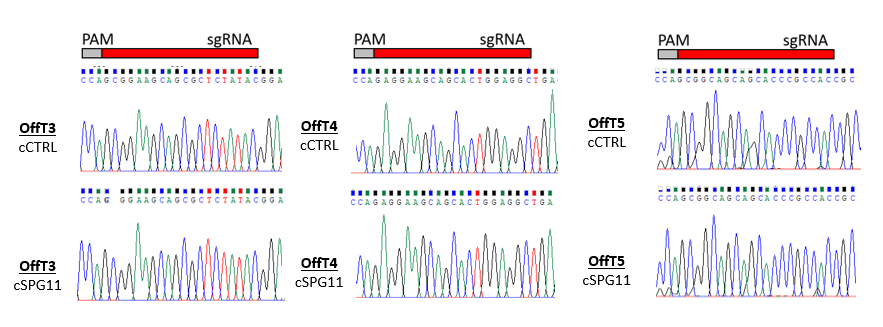

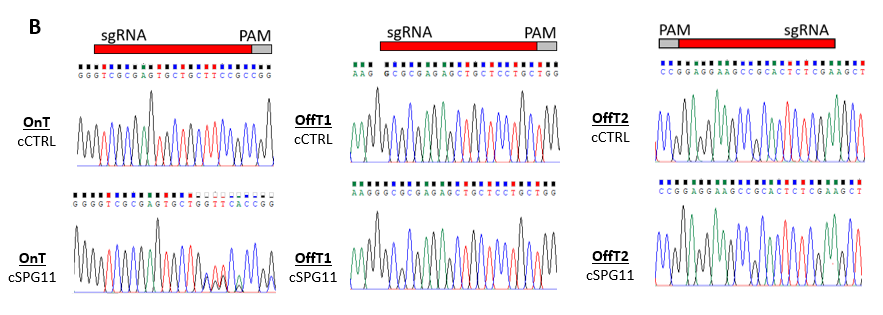


Fig. S3 Off-target analysis of the sgRNA92 used in the genome editing process. (A) Table containing the sgRNA sequence used for exon1 targeting and the potential off-target sequences at exonic regions as determined by CRISPOR web-tool (<http://crispor.tefor.net/>). (B) Sequences of on-target (OnT) and off-target (OffT) regions acquired by Chromas software (<https://technelysium.com.au/wp/chromas/>) following genomic DNA sequencing of cSPG11 and cCTRL.

Table S1 - primer list

| Primer | Primer sequence 5’🡪 3’ | Application |
| --- | --- | --- |
| Ctip2 Fw | GAGTACTGCGGCAAGGTGTT | qPCR |
| Ctip2 Rw | TAGTTGCACAGCTCGCACTT | qPCR |
| VGLUT1 Fw | TTTTCTGGGGCTACATTGTCAC | qPCR |
| VGLUT1 Rv | ACTCCGTTCTAAGGGTGGGG | qPCR |
| OnT (SPG11 exon1) Fw | AGCTGGACCAAGAAGGCAAA | genotyping PCR |
| OnT (SPG11 exon1) Rv | GAAAGAATCAGCGCCTCAGC | genotyping PCR |
| OffT1 (VWA2) Fw | ATGGCATTCCCTTCCGTGG | genotyping PCR |
| OffT1 (VWA2) Rv | CTGGTGCCTGAAAGAGTGGG | genotyping PCR |
| OffT2 (ZKSCAN2) Fw | GCACCTTACCATTTGGGCTCTG | genotyping PCR |
| OffT2 (ZKSCAN2) Rv | CAAGCCTGTCATCGGAAGAGC | genotyping PCR |
| OffT3 (CYP27A1) Fw | AAGGGAAACTGAGGCACAC | genotyping PCR |
| OffT3 (CYP27A1) Rv | GAGGAGAGGGAGCACAACCT | genotyping PCR |
| OffT4 (PLEKHG4) Fw | CCAGCGAGGAAGCCATCAAC | genotyping PCR |
| OffT4 (PLEKHG4) Rv | CAAATGGCACTCACACAGGGT | genotyping PCR |
| OffT5 (ATXN7) Fw | GCGGGAGTCGAAAGCGAAAG | genotyping PCR |
| OffT5 (ATXN7) Rv | CTCAACCCACAGATTCCACGAC | genotyping PCR |

***Supplemental experimental procedures***

Western blot

*Protein sample preparation*

The cells were harvested on ice and resuspended in TSE buffer (Tris 1M Tris, EDTA 0.5M, NaCl 2M ) supplemented with phosphatase and protease inhibitors), the cells were sonicated at 65% amplitude 5 cycles of 30 seconds. Following an addition of Triton to an end concentration of 1%, the cells were incubated on ice for 30 minutes. Afterwards, the homogenates were centrifugated at 14,000 rpm for 10 minutes at 4^0^C. For immunoblotting, 30 µg to protein was separated by SDS-PAGE and blotted on PVDF membrane. Primary anti-SPG11 antibody (1:500, Proteintech) was incubated at 4ºC over-night in Tris buffered saline and 0.1 % Tween-20 supplemented with 3 % bovine serum albumin. The blots were then incubated with secondary antibodies conjugated to horseradish peroxidase and subsequently developed by chemical luminescence.
